# From the Gut to the Brain: Microplastic‐Associated Neurovascular Dysfunction and Implications for Stroke Risk

**DOI:** 10.1002/advs.202520278

**Published:** 2026-01-12

**Authors:** Hongxing Wang, Yujiao He, Pei Zou, Xiaoyi Wang, Gang Peng, Xiangying Deng

**Affiliations:** ^1^ Department of Rehabilitation School of Medicine Chongqing University Three Gorges Hospital Chongqing University Chongqing China; ^2^ Institute of Medical Sciences National Clinical Research Center for Geriatric Disorders Xiangya Hospital Central South University Changsha Hunan China; ^3^ Xinjiang Key Laboratory of Neurological Disorder Research The Second Affiliated Hospital of Xinjiang Medical University Urumgi China; ^4^ Department of Neurosurgery Xiangya Hospital Central South University Changsha Hunan China; ^5^ Department of Bone and Joint Rehabilitation Xiangya Boai Rehabilitation Hospital Changsha Hunan China

**Keywords:** blood‐brain barrier, gut‐brain axis, microplastics, neurovascular dysfunction, stroke susceptibility

## Abstract

Microplastics (MPs) have emerged as pervasive environmental contaminants with increasing relevance to neurovascular health. Following oral exposure, accumulating evidence suggests that MPs can disrupt gut microbial homeostasis, impair intestinal epithelial barrier integrity, and engage the gut‐brain axis (GBA), thereby promoting systemic and central inflammatory responses. These interconnected processes are linked to blood‐brain barrier (BBB) dysfunction, cerebral microvascular impairment, and neurovascular alterations that are biologically relevant to stroke susceptibility. Evidence derived largely from animal and in vitro models, together with emerging epidemiological observations, supports the biological plausibility that microplastic (MP) exposure may contribute to neurovascular vulnerability through mechanisms involving endothelial inflammation, pro‐thrombotic signaling, and atherosclerotic progression. However, substantial heterogeneity in exposure paradigms, particle characteristics, and analytical methodologies limits direct causal inference and translational interpretation in humans. Future research should prioritize standardized exposure frameworks and integrate multi‐omics approaches with artificial intelligence (AI)‐assisted analysis to better define exposure–response relationships and mechanistic pathways underlying MP‐associated cerebrovascular alterations. Such efforts are essential for improving risk assessment and informing evidence‐based strategies for environmental neurovascular health.

## Graphical abstract

Chronic oral exposure to microplastics may disrupt gut microbiota homeostasis and intestinal barrier integrity, potentially engaging the gut–brain axis and systemic inflammatory responses. These alterations may be associated with impaired blood–brain barrier function, cerebral microvascular dysfunction, and enhanced endothelial inflammation, pro‐thrombotic signaling, and atherosclerotic processes, collectively contributing to increased neurovascular vulnerability and stroke risk.

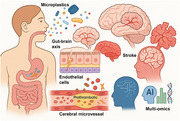

## 1 Introduction

Plastics have become integral to modern life, and their massive production, widespread use, and gradual degradation have led to pervasive MP contamination—now regarded as an emerging threat to environmental and public health [1]. MPs, defined as synthetic polymer particles <5 mm, encompass a wide spectrum of sizes, shapes, and chemical compositions, originating from both intentionally manufactured primary MPs and the fragmentation of larger plastic materials [2]. Common polymers such as polyethylene (PE), polypropylene (PP), polystyrene (PS), polyvinyl chloride (PVC), and polyethylene terephthalate (PET), along with additives including plasticizers, flame retardants, stabilizers, and dyes, impart complex physicochemical properties that govern their environmental persistence, biological reactivity, and interactions with cellular membranes [2, 3, 4]. Notably, the high surface area and diverse functional groups of MPs enable them to adsorb heavy metals, persistent organic pollutants, pesticides, pharmaceuticals, and other contaminants, forming pollutant‐laden “complexes” that often demonstrate enhanced bioavailability and synergistic toxicity within biological systems [1, 4, 5].

Among the various exposure routes, oral ingestion represents the predominant pathway for human MP exposure, with MPs widely detected in drinking and bottled water, seafood, salt, and dairy products [6, 7]. After entering the gastrointestinal (GI) tract, MPs can disrupt epithelial tight junctions, increase intestinal permeability, and induce gut microbiota dysbiosis and chronic inflammation [8, 9]. These alterations facilitate the systemic translocation of MPs and their associated pollutants, allowing their accumulation in distal organs and activation of the GBA [10, 11]. Accumulating in vivo and in vitro evidence further indicates that MPs can cross biological barriers—including the BBB—activate microglia, trigger oxidative stress and inflammatory cascades, and compromise cerebral microvascular integrity [12, 13, 14]. Such neurovascular disturbances are particularly relevant to stroke, a leading global cause of mortality and disability, in which chronic inflammation, vascular dysfunction, and BBB breakdown are key pathological drivers.

Given these emerging insights, this review synthesizes current knowledge on MP translocation following GI exposure and delineates the inflammatory and neurovascular pathways through which MPs may contribute to stroke onset and progression. We place particular emphasis on intestinal barrier dysfunction, GBA‐mediated signaling, BBB permeability alterations, and cerebral endothelial impairment. By integrating perspectives from environmental toxicology and neurovascular biology, we also identify critical knowledge gaps and propose future research priorities essential for establishing early‐warning frameworks and precision strategies to mitigate MP‐induced neurotoxicity.

## 2 Overview and Ingestion Routes of MPs

### 2.1 Human Exposure Pathways Most Relevant to GBA‐Mediated Effects

Among all human exposure routes, oral ingestion is the predominant and most biologically relevant pathway through which MPs influence the GBA. MPs have been widely detected in drinking water, seafood, salt, dairy products, and various plant‐based foods, making daily ingestion virtually unavoidable [8, 15, 16]. After entering the gastrointestinal tract, MPs come into direct contact with the intestinal epithelium, and their uptake and systemic distribution are determined by factors such as particle size, surface chemistry, and polymer composition. Particles <10 µm can penetrate the epithelial barrier—especially under conditions of increased gut permeability—via M‐cell–mediated persorption or active transcytosis, thereby entering the bloodstream or lymphatic system and reaching distal organs including the brain [13]. MPs retained within the mucus layer may induce chronic mucosal inflammation and gut microbiota dysbiosis, both of which profoundly modulate neural, immune, and endocrine signaling along the GBA [17]. Inhalation contributes mainly indirectly, as most airborne MPs deposited on the respiratory tract are subsequently cleared and swallowed, increasing gastrointestinal burden rather than entering through primary pulmonary uptake. Dermal exposure is negligible and is not considered relevant to GBA‐related toxicity.

Importantly, MPs in the gastrointestinal environment function not only as particles but also as carriers of co‐adsorbed pollutants, such as heavy metals, antibiotics, and endocrine‐disrupting chemicals, forming “MP‐contaminant complexes” with enhanced bioavailability and synergistic toxicity [18, 19]. MPs can enrich hydrophobic pollutants, alter their desorption kinetics, and reduce degradation, thereby increasing their biological accessibility. Once bound to MPs, pollutant entry into cells is no longer limited to passive diffusion; rather, pollutants can be co‐internalized via MP‐mediated endocytosis, substantially enhancing cellular uptake efficiency [20]. These complexes typically induce stronger toxic effects than pollutants alone, including elevated ROS production, membrane disruption, mitochondrial dysfunction, protein‐corona alterations, and amplified inflammatory signaling. Polymer type, particle size, surface charge, and aging status further modulate adsorption capacity and downstream toxicological outcomes. Growing evidence demonstrates that MP‐bound pollutants cause more severe neurovascular injury, endothelial barrier disruption, and inflammatory cascade amplification than free pollutants [21, 22], highlighting the need to consider MPs as active vectors that alter the fate and toxicity of environmental contaminants in health risk assessment.

Overall, these findings confirm oral ingestion as the primary and most biologically impactful route through which MPs affect the gut–brain axis. MPs not only cross the intestinal barrier and disrupt mucosal and microbial homeostasis but also act as active carriers of co‐adsorbed pollutants, forming highly toxic MP–contaminant complexes. This dual‐risk model—combining particle effects and pollutant transport—significantly amplifies neurovascular and systemic toxicity, underscoring the need to incorporate MP–pollutant interactions into future mechanistic and risk‐assessment frameworks.

### 2.2 Systemic Translocation of MPs

#### 2.2.1 Tight‐Junction Disruption and Barrier Failure

**FIGURE 1:**
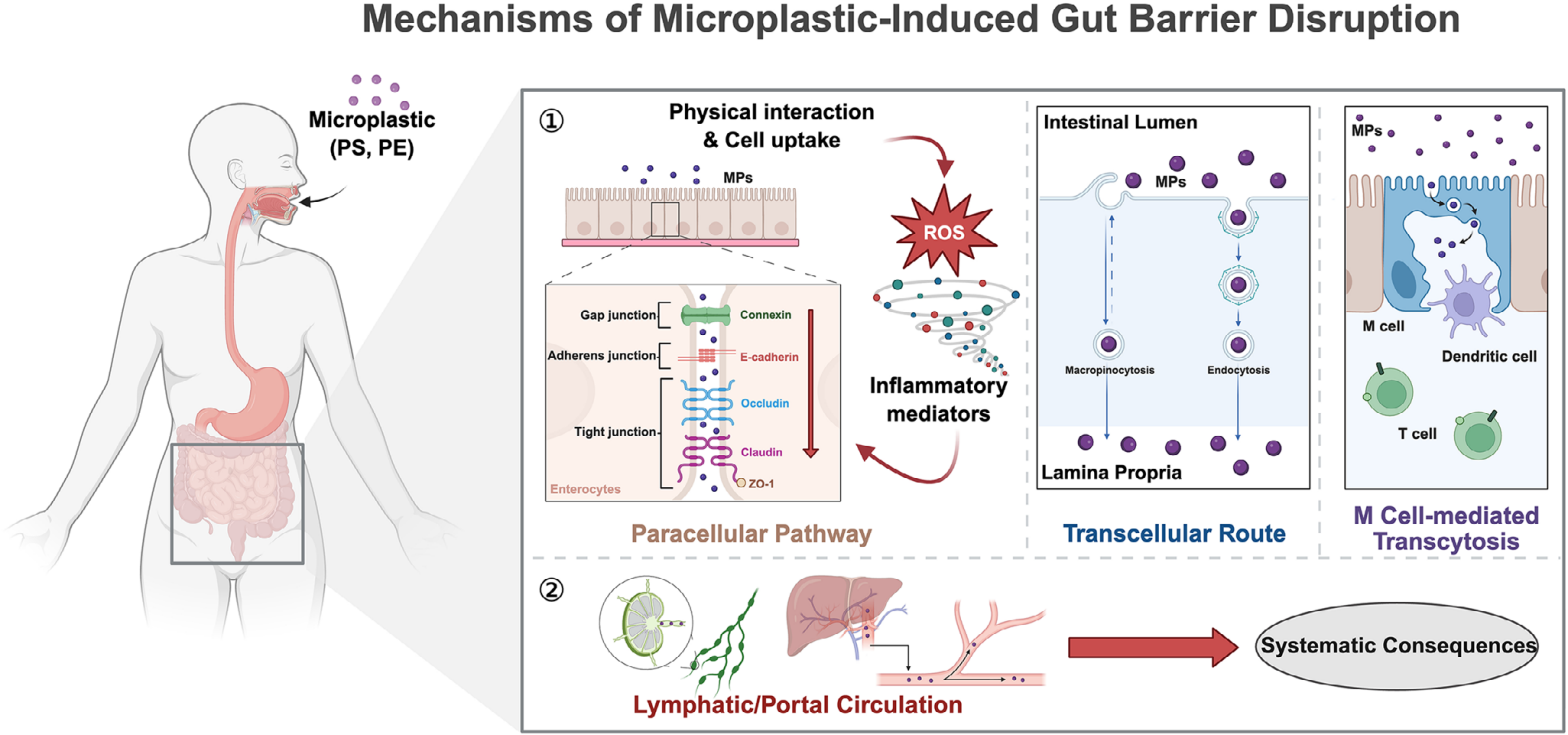
MP‐induced disruption of the intestinal barrier and systemic translocation. MPs entering the gastrointestinal tract induce ROS production and activate inflammatory pathways such as NF‐κB and MAPK, resulting in mucus thinning and downregulation of tight‐junction proteins (claudin, occludin, ZO‐1). The resulting barrier permeability allows MPs to cross the epithelium via paracellular leakage, transcellular uptake, or M‐cell–mediated transport, subsequently reaching the lamina propria and entering systemic circulation through lymphatic or portal routes, thereby promoting systemic inflammation and potentially contributing to neurovascular dysfunction. Created with BioRender.

MPs can induce a “leaky‐gut” state by directly damaging intestinal tight junctions (claudins, occludin, ZO‐1) and activating inflammatory pathways such as NF‐κB and MAPK [8, 23]. These signals enhance the release of cytokines (IL‐6, TNF‐α), destabilize junctional complexes, and promote epithelial contraction, collectively widening paracellular spaces and increasing intestinal permeability. Concurrent oxidative stress further accelerates tight‐junction degradation. Thus, MP‐induced barrier dysfunction arises through both physical irritation and molecular signaling, establishing the primary prerequisite for systemic translocation (Figure 1).

#### 2.2.2 Microbiota Dysbiosis and Mucus Layer Impairment

Beyond structural damage, MPs alter gut microbial ecology by reducing beneficial genera (e.g., Lactobacillus, Bifidobacterium) and depleting short‐chain fatty acid–producing commensals [7, 24]. At the same time, MPs impair goblet cell function, thin the mucus layer, and promote mucosal oxidative stress. This combined dysbiosis–mucus weakening amplifies the leaky‐gut phenotype, heightens local immune activation, and creates a permissive environment allowing MPs to penetrate deeper into the lamina propria (Figure 1).

#### 2.2.3 Translocation Routes and Systemic Dissemination

Once the epithelial barrier is compromised, MPs—particularly those <10 µm—can translocate via multiple pathways, including paracellular leakage through disrupted tight junctions, active transcellular endocytosis and basolateral extrusion, and M‐cell–mediated persorption. After entering the lamina propria, MPs access systemic circulation through mesenteric lymphatics, portal blood flow, or chylomicron‐associated transport. Animal studies confirm their accumulation in the liver, kidneys, lungs, and even the brain [11]. Systemically distributed MPs can trigger chronic inflammation and oxidative stress, providing a mechanistic link between GI barrier dysfunction and neurovascular injury, particularly in populations with increased BBB vulnerability (Figure 1).

#### 2.2.4 Physicochemical Determinants of MP Translocation and Neurovascular Toxicity

Current evidence indicates that the in vivo translocation potential and neurovascular toxicity of MPs are not fixed properties but are highly dependent on specific exposure contexts [13, 25, 26]. At the particle‐size level, nanoscale MPs are more likely to traverse the intestinal barrier or the BBB under conditions of barrier impairment, subsequently accumulating in brain tissue and inducing oxidative stress and microvascular injury. In contrast, larger particles tend to remain confined within the intestinal lumen and exert limited direct effects on systemic target organs. Moreover, when epithelial barriers remain structurally intact, exposure levels approximate real‐world environmental concentrations, or surface charge and protein‐corona characteristics do not favor translocation, the tissue accumulation and biological effects of MPs are often minimal—highlighting the strong context dependence of their in vivo behavior. Polymer composition and particle morphology further shape biological reactivity: distinct protein corona profiles formed by different polymers influence cellular uptake efficiency and inflammatory activation, while fibrous or rough‐surfaced particles generally exhibit greater membrane‐disruptive and immunostimulatory potential than spherical particles. These differences help explain why high‐dose studies frequently report inflammation, oxidative stress, and barrier disruption, whereas a substantial proportion of environmentally relevant exposure studies observe little to no detectable toxicity. Collectively, these findings suggest that MP toxicity likely exhibits dose thresholds and is modulated by physicochemical properties and exposure conditions. Therefore, a balanced evaluation that integrates both positive and null findings is essential to avoid biased interpretations of the neurovascular risks associated with MP exposure.

In summary, the systemic translocation of MPs arises from gut barrier disruption, microbiota–mucus layer impairment, and particle‐specific physicochemical properties, allowing especially <10 µm particles to enter circulation and drive chronic inflammation and neurovascular injury. Because toxicity varies with particle size, polymer type, protein‐corona composition, and exposure dose—explaining discrepancies between high‐dose and environmentally relevant studies—neurovascular risk assessment must integrate both positive and null findings to avoid biased conclusions.

## 3 The Impact of MPs on the GBA

### 3.1 Gut Dysbiosis

**FIGURE 2:**
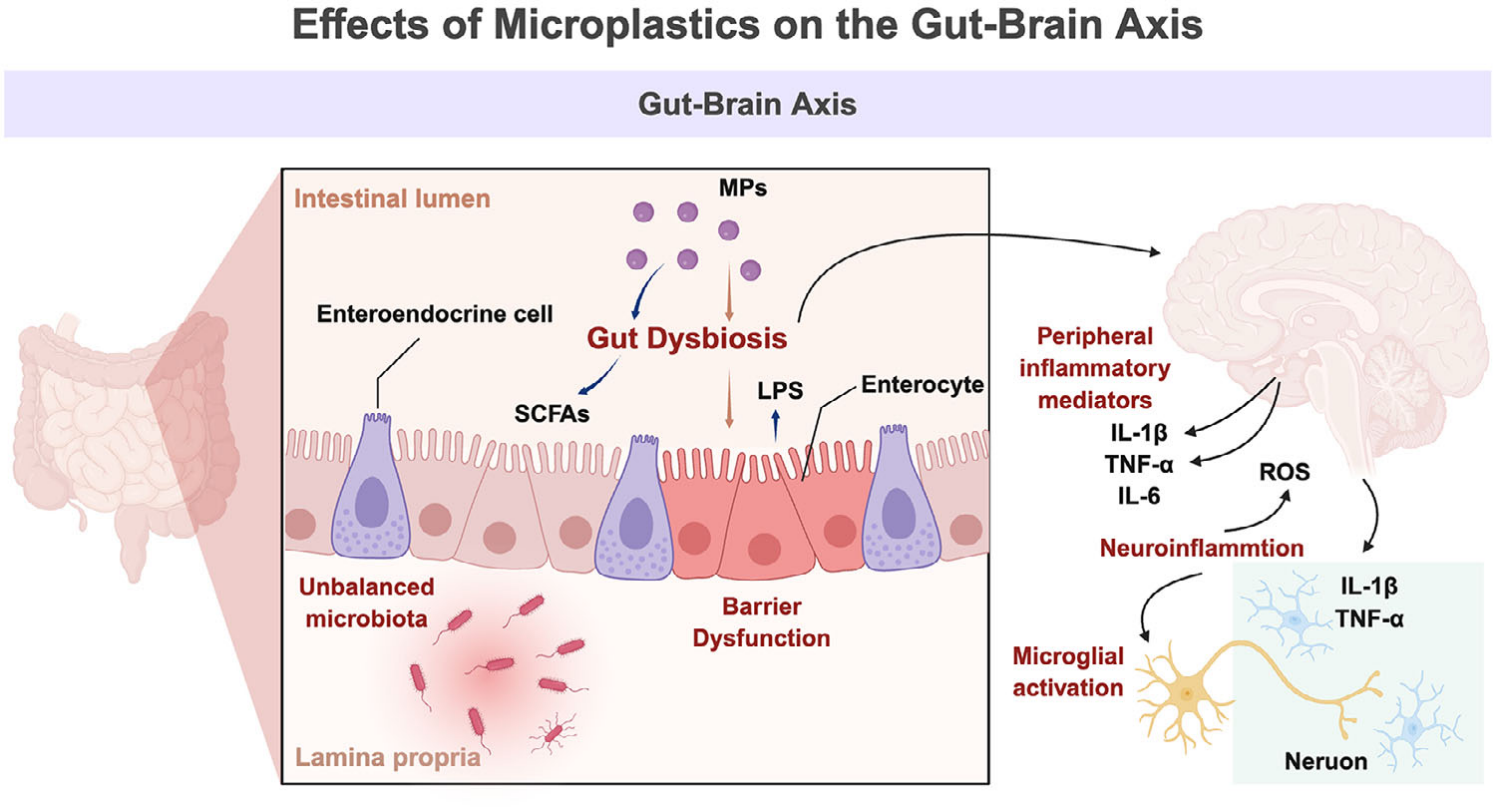
Mechanisms by which MPs disrupt the GBA. Ingested MPs disturb gut microbiota balance by reducing beneficial genera (e.g., Lactobacillus, Bifidobacterium, Akkermansia) and enriching pro‐inflammatory bacteria (e.g., Proteobacteria, Escherichia–Shigella), leading to decreased SCFAs and elevated LPS. These changes impair mucus secretion and tight‐junction proteins (ZO‐1, occludin, claudin‐1), producing a “leaky‐gut” state and systemic inflammation. Circulating cytokines (IL‐6, TNF‐α, IL‐1β) and LPS activate the TLR4/NF‐κB and MAPK pathways, damage the blood–brain barrier, and trigger microglial M1 polarization and neuroinflammation via humoral and vagal‐nerve routes, collectively increasing stroke susceptibility. Created with BioRender.

Upon entering the GI tract, MPs disrupt not only the structural integrity of the intestinal epithelium but also the ecological stability of the gut microbiota—an essential regulator of GBA homeostasis (Figure 2). The gut microbiome is critical for barrier maintenance, metabolic regulation, and immune balance, and its diversity is tightly linked to healthy neuro–immune communication [27]. Accumulating animal evidence shows that exposure to MPs of different sizes and doses (e.g., PS particles) markedly reduces microbial α‐diversity and reshapes community composition [28]. Beneficial taxa such as *Lactobacillus*, *Bifidobacterium*, and *Akkermansia* consistently decline, while facultative anaerobes and pathobionts—including *Proteobacteria* and *Escherichia–Shigella—*increase [29, 30, 31]. This dysbiotic shift weakens mucosal resilience, amplifies local inflammation, and may propagate systemic and neurovascular disturbances. Importantly, MP‐induced microbiota alterations are both persistent and dose‐dependent, with even short‐term exposure capable of establishing long‐lasting microbial imbalance [32].

Changes in microbial composition further disrupt functional metabolites, intensifying systemic inflammation. Short‐chain fatty acids (SCFAs)—including acetate, propionate, and butyrate—are key fermentation products of commensal bacteria and are essential for maintaining epithelial integrity, regulating immune responses, and exerting anti‐inflammatory effects [33, 34]. However, MP exposure models consistently report reduced SCFA levels, particularly a marked decrease in butyrate, a critical mucoprotective metabolite [35]. In parallel, intestinal concentrations of lipopolysaccharide (LPS), a potent Gram‐negative endotoxin, are significantly elevated. LPS readily crosses a compromised intestinal barrier, entering systemic circulation and inducing “metabolic endotoxemia” [36]. Through activation of the TLR4/NF‐κB pathway, LPS promotes excessive production of TNF‐α and IL‐1β, establishing a chronic inflammatory milieu that destabilizes cerebrovascular homeostasis and impairs neural function [37]. Notably, LPS can itself traverse the BBB and directly activate microglia and astrocytes within the Central Nervous System (CNS), initiating robust neuroinflammatory responses [38] (Figure 2). Thus, gut dysbiosis functions not only as a local disturbance but also as an “amplifier” and “relay station” that propagates MP‐induced systemic and neurovascular toxicity.

Collectively, MP‐induced gut microbiota dysbiosis weakens the mucosal barrier and amplifies systemic inflammation by reducing beneficial bacteria, disrupting key metabolites such as SCFAs, and elevating gut‐derived LPS. LPS further activates the TLR4/NF‐κB pathway and enters the CNS to trigger glial cell activation, making gut dysbiosis a central amplifying mechanism that drives neuroinflammation and neurovascular injury.

### 3.2 Disruption of Intestinal Barrier Function

Upon entry into the GI tract, MPs rapidly disrupt the two principal layers of the intestinal physical barrier—the mucus layer and epithelial tight junctions. Under physiological conditions, the goblet cell–derived mucin network (e.g., MUC2) forms a highly organized protective matrix that prevents direct contact of luminal pathogens, toxins, and exogenous particles with the epithelium [39]. Emerging in vivo studies, however, reveal that MP exposure leads to pronounced mucus impairment, including reduced goblet cell abundance, diminished mucin secretion, and marked thinning or focal loss of the mucus layer [8, 10, 18, 40]. These alterations significantly heighten epithelial susceptibility to luminal insults. In parallel, MPs compromise epithelial integrity by destabilizing tight‐junction complexes. In PS‐MP‐exposed murine models, occludin, claudin‐1, and ZO‐1 are consistently downregulated, accompanied by widened paracellular spaces and severe architectural disorganization on ultrastructural examination [8] (Figure 2). This coordinated erosion of the mucus layer and tight junctions signals a collapse of barrier homeostasis—a hallmark “leaky‐gut” phenotype. Such barrier failure permits the translocation of luminal toxins, inflammatory mediators, and microbial products into the lamina propria, triggering early systemic immune activation and setting the stage for downstream neurovascular perturbations associated with MP exposure.

Concurrent with structural damage, MPs also activate the intestinal immune system, driving the upregulation and sustained release of pro‐inflammatory mediators that further destabilize the epithelial barrier. In vivo studies show that MP exposure markedly elevates IL‐1β, TNF‐α, and IL‐6 levels in intestinal tissues, accompanied by activation of the NF‐κB pathway and increased ROS generation [37]. These inflammatory cascades accelerate tight‐junction degradation, promote epithelial apoptosis, and impair the basement membrane, establishing a self‐amplifying cycle of inflammation and barrier failure. The resulting pro‐inflammatory microenvironment facilitates the recruitment of macrophages and dendritic cells into the lamina propria, where additional cytokines and chemokines are released, further disrupting intestinal homeostasis [41, 42]. Importantly, these locally generated inflammatory signals can propagate systemically—via hematogenous or neural pathways—to distal organs. Experimental data indicate that such signals impair cerebral microvascular endothelial function and increase BBB permeability [43]. Collectively, MP‐induced intestinal inflammation represents not merely a localized insult but a potential initiating event for systemic immune activation and neurovascular dysfunction, functioning as a critical “first hit” in the pathogenesis of complex disorders such as stroke.

Taken together, MPs rapidly erode the intestinal mucus layer and tight‐junction integrity while activating mucosal immune pathways, creating a “leaky‐gut” state characterized by elevated IL‐1β, TNF‐α, IL‐6, NF‐κB activation, and ROS accumulation. This locally amplified inflammation facilitates systemic dissemination of toxic and immune signals, which can impair cerebral endothelium and increase BBB permeability, positioning gut barrier collapse as a critical initiating step in MP‐induced neurovascular dysfunction.

### 3.3 GBA‐Mediated Neuroinflammation

**FIGURE 3:**
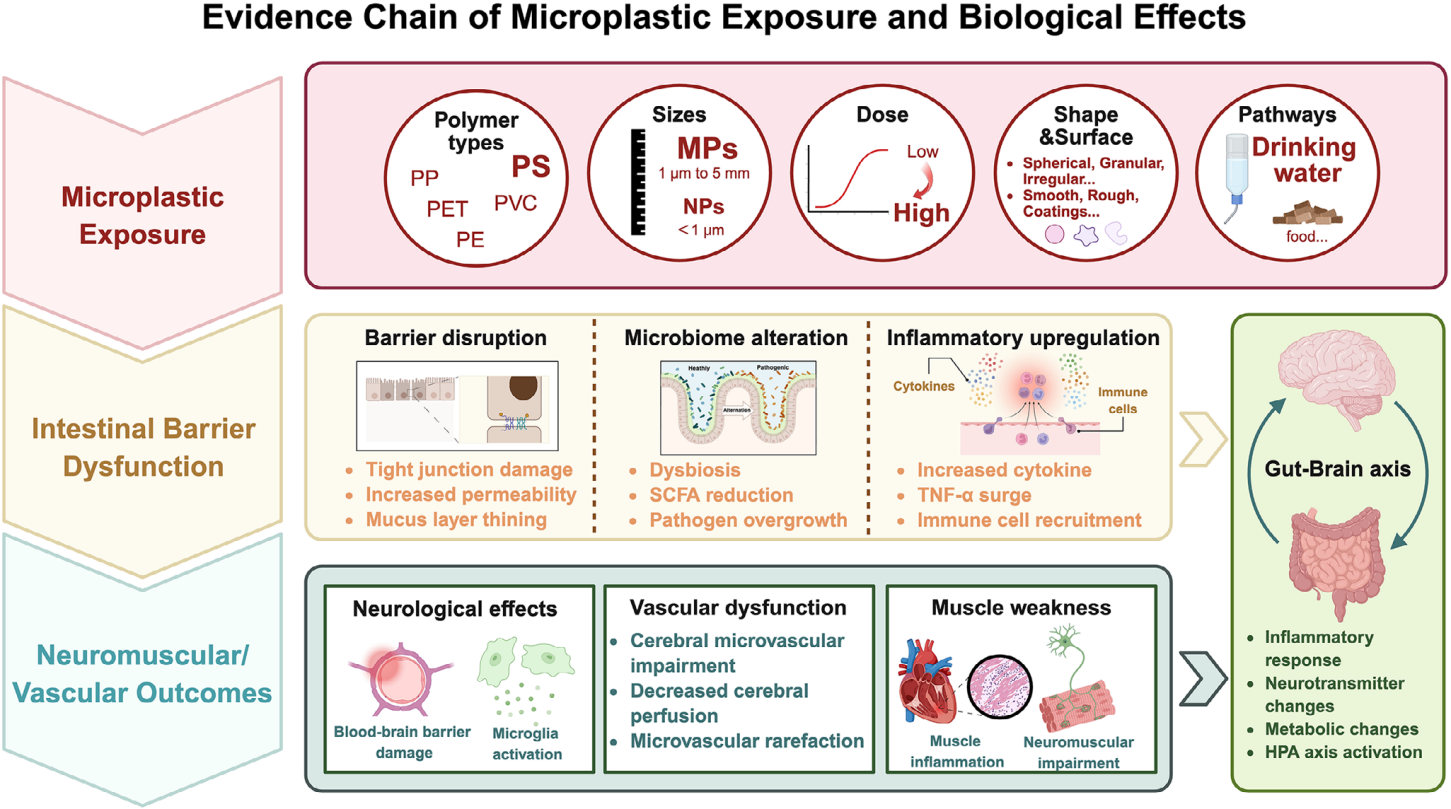
Mechanisms by which ingested MPs induce GBA disruption and neurovascular/ neuromuscular injury. Micro‐ and nanoplastics present in drinking water and food enter the gastrointestinal tract, where they disrupt the intestinal mucosal barrier, downregulate tight‐junction proteins, thin the mucus layer, and induce gut microbiota dysbiosis, collectively leading to elevated local and systemic inflammation (IL‐6, TNF‐α, IL‐1β). These gut‐derived inflammatory signals propagate through the gut–brain axis to affect the central nervous system, causing increased blood–brain barrier permeability, microglial activation, impaired cerebral microvascular regulation, and peripheral neuromuscular dysfunction, ultimately heightening the risk of neurovascular injury. Created with BioRender.

The GBA, a bidirectional communication network integrating neural, immune, and endocrine pathways, provides a major route through which gastrointestinal disturbances influence CNS function [44, 45]. MP‐induced gut dysbiosis and barrier disruption consistently elevate peripheral cytokines such as IL‐6, TNF‐α, and IL‐1β, which may penetrate the BBB or act on endothelial cells, astrocytes, and microglia to initiate neuroinflammatory cascades [14] (Figure 3). Sustained peripheral inflammation further promotes ROS release and chemokine production by circulating immune cells, exacerbating BBB impairment and increasing cerebral vulnerability to toxins and inflammatory signals [46].

In mice and *C. elegans* models, low‐dose MP exposure accelerates dopaminergic neurodegeneration and motor dysfunction, largely through disruption of mucosal, immune, and microbial barriers, thereby inducing PD‐like neuronal pathology. Notably, even non‐penetrating MPs can amplify neuroinflammation by triggering excessive ROS accumulation and persistent mitochondrial unfolded protein response [47]. Recent evidence further indicates that short‐term PS‐nanoplastic (PS‐NP) exposure during early adulthood can exert long‐lasting neurotoxic effects later in life. Months after exposure, animals exhibit cognitive deficits and anxiety‐like behavior, accompanied by prolonged retention of PS‐NPs in the brain. The resulting neuroinflammation does not spontaneously resolve but is amplified through microglia–astrocyte feedback loops, driving synaptic loss and neuronal degeneration. In vitro data show that neurovascular unit cells internalize PS‐NPs through multiple endocytic pathways, making clearance difficult once they cross the BBB. These findings collectively highlight the latent and persistent neurotoxicity of PS‐NPs, while suggesting that modulation of microglial overactivation may offer a potential therapeutic strategy [48]. Moreover, studies consistently demonstrate robust microglial activation and heightened NF‐κB and MAPK signaling in MP‐exposed animals, supporting a cross‐barrier mechanism whereby gut‐derived inflammation propagates to the CNS [49, 50]. Together, these observations provide a mechanistic basis for how MPs may drive neuroinflammation, cognitive impairment, and cerebrovascular dysfunction, ultimately contributing to increased stroke susceptibility.

In addition to humoral signaling, the vagus nerve—one of the principal neural conduits of the GBA—plays a pivotal role in transmitting MP‐induced inflammatory cues to the CNS. Composed of ∼80% afferent fibers, the vagus nerve continuously monitors intestinal physiological states and relays perturbations to central hubs such as the brainstem, limbic system, and hypothalamus [51, 52]. Under MP exposure, intestinal barrier disruption and microbiota dysbiosis elevate peripheral inflammatory mediators including IL‐1β, TNF‐α, and LPS, which can be sensed by Toll‐like receptors and cytokine receptors expressed on vagal afferent terminals [9, 53]. These signals converge in the nucleus tractus solitarius (NTS) and are subsequently propagated to the parabrachial nucleus, hypothalamus, and limbic circuits, thereby modulating central inflammatory thresholds.

Vagal signaling also interacts with the cholinergic anti‐inflammatory pathway, particularly through the α7nAChR axis, which regulates microglial activation states and biases microglia toward a pro‐inflammatory phenotype when gut‐derived inflammatory inputs are strong. The integration of vagal neural transmission with circulating inflammatory cytokines synergistically amplifies microglial NF‐κB activation, NOD‐like receptor family pyrin domain‐containing 3 (NLRP3) inflammasome priming, and ROS accumulation, exacerbating neuroinflammation and neuronal vulnerability [43, 54, 55]. Consistently, experimental inhibition of vagal activity—pharmacologically or surgically—attenuates CNS inflammation in multiple neurodegenerative and cerebrovascular models [56, 57, 58], underscoring the vagus nerve as a critical bidirectional communication axis through which MPs propagate peripheral inflammatory signals to the brain. Together, these humoral and neural pathways provide a dual‐channel mechanism by which MPs may amplify neuroinflammation and increase susceptibility to stroke.

Overall, MPs amplify gut–brain axis signaling by inducing gut dysbiosis, barrier disruption, and systemic inflammation, with elevated cytokines and LPS propagating across the BBB or via vagal afferents to activate microglia, trigger NF‐κB/MAPK/NLRP3 pathways, and promote persistent neuroinflammation and neurodegeneration. Through this dual humoral–neural mechanism, MPs drive cognitive impairment and cerebrovascular dysfunction, ultimately heightening susceptibility to stroke.

## 4 MP‐Induced Neurovascular Dysfunctions

### 4.1 Alterations in Blood‐Brain Barrier Permeability

The BBB is one of the most essential structural and functional barriers of the CNS, maintaining neural homeostasis by tightly regulating molecular entry into the brain [59]. It is primarily composed of brain microvascular endothelial cells (BMECs), a basement membrane, pericytes, and astrocytic end‐feet, with its selective permeability governed by tight‐junction proteins such as claudin‐5, occludin, and ZO‐1 [60]. Emerging evidence indicates that once MPs enter systemic circulation, they may subsequently reach and penetrate the BBB. Notably, nanoplastics (NPs <100 nm) demonstrate a strong capacity to cross BMECs through transcellular transport pathways [61]. In vivo studies report that PS MP exposure induces marked BMEC apoptosis, microvascular disruption, and neuroinflammatory activation [62]. Consistently, in vitro experiments reveal that MPs directly impair BMEC function by triggering mitochondrial dysfunction and apoptotic signaling, thereby reducing endothelial viability and compromising tight‐junction integrity [22, 63, 64]. The experimental results further indicate that PS‐MP–induced injury to BMECs exhibits clear dose dependence and polymer specificity: higher exposure levels or certain polymer types more readily lead to reduced cell viability and membrane integrity disruption. BMEC apoptosis typically emerges 12–24 h after exposure, accompanied by increased barrier permeability and disassembly of tight‐junction structures. At the molecular level, classical neuroinflammatory pathways—including NF‐κB, MAPK, and NLRP3—are activated, promoting the release of pro‐inflammatory cytokines and the accumulation of oxidative stress, thereby exacerbating microvascular dysfunction. Collectively, these findings indicate that BMEC injury arises from the synergistic effects of dosage, material properties, and inflammatory signaling rather than from a single mechanistic driver. Such BBB impairment weakens its exclusionary capacity and facilitates the entry of peripheral cytokines and circulating toxins into the CNS, ultimately amplifying neurovascular injury.

**FIGURE 4:**
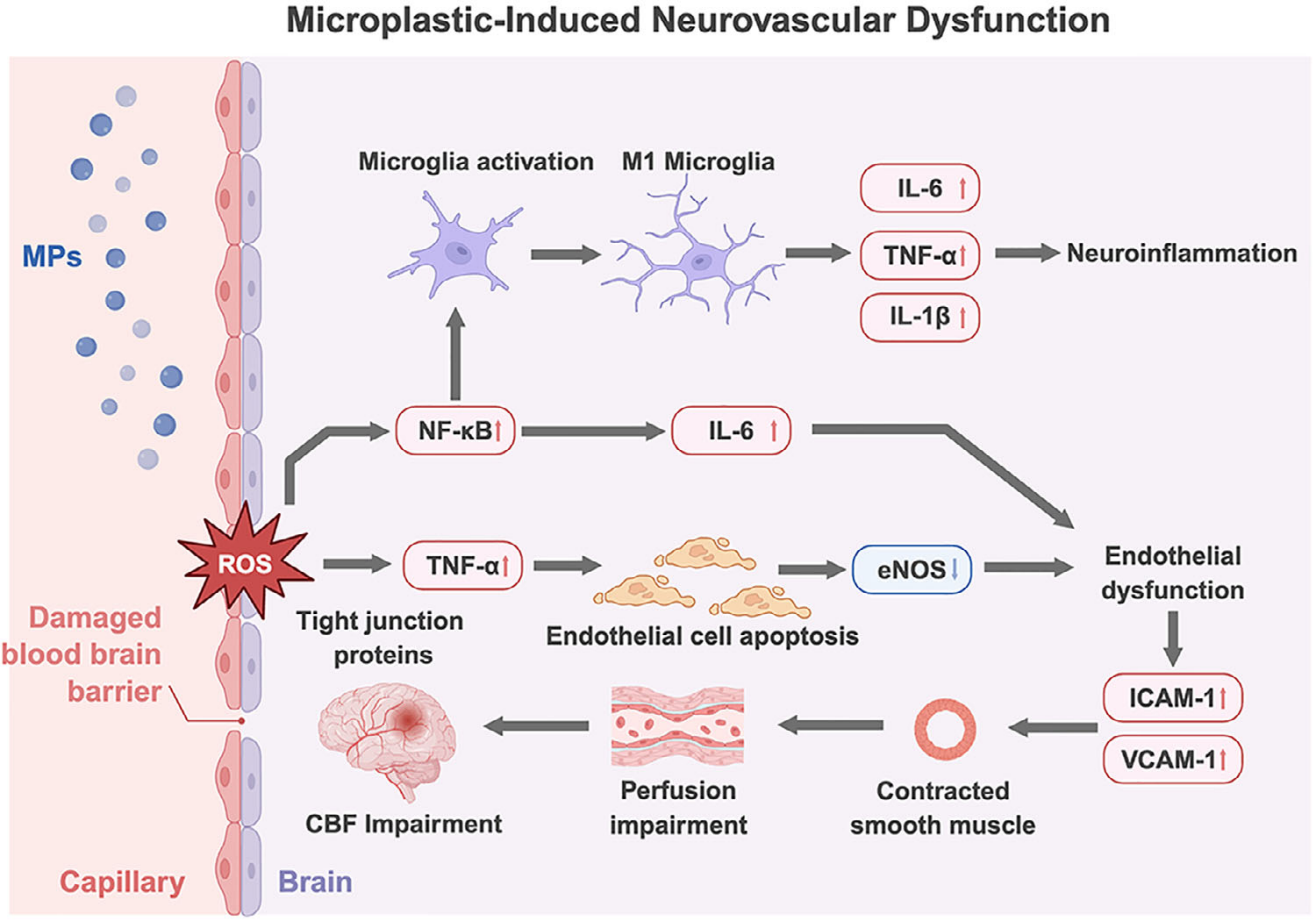
Neurovascular dysfunction induced by MPs exposure. MPs entering circulation can cross the BBB and directly damage BMECs, leading to mitochondrial injury, tight‐junction disruption (claudin‐5, ZO‐1, occludin), and increased BBB permeability. Excessive ROS generation activates the NF‐κB pathway, promoting inflammation (TNF‐α, IL‐6) and endothelial apoptosis. Within the brain, MPs and inflammatory cytokines trigger microglial M1 polarization and neuroinflammation, releasing IL‐1β, IL‐18, NO, and ROS, which further injure neurons and astrocytes. Endothelial dysfunction (eNOS), up‐regulated ICAM‐1/VCAM‐1, and impaired vascular autoregulation together result in cerebral microvascular constriction, perfusion imbalance, and stroke susceptibility. Created with BioRender.

At the molecular level, oxidative stress has emerged as a central driver of MP‐induced BBB disruption. MP exposure markedly elevates ROS levels in brain tissue and activates pro‐inflammatory signaling cascades—most prominently the NF‐κB pathway—which in turn upregulates cytokines such as TNF‐α and IL‐6 [65, 66, 67] (Figure 4). Beyond amplifying inflammation, NF‐κB also regulates the transcriptional suppression and proteasomal degradation of tight‐junction proteins [68]. Consistently, MP‐exposed animal models exhibit pronounced downregulation of claudin‐5, occludin, and ZO‐1, leading to widened inter‐endothelial gaps and increased paracellular permeability [69, 70]. ROS‐driven Ca^2^^+^ influx and cytoskeletal remodeling further destabilize endothelial junctions, exacerbating BBB failure [71].

At the same time, MPs and their adsorbed substances can synergistically impair BBB integrity through additional molecular pathways. MP‐induced ROS activates not only NF‐κB but also MAPK signaling, promoting tight‐junction ubiquitination and degradation [72]. MPs carrying LPS strongly activate the TLR4–MyD88–IRAK–TRAF6 cascade, driving NF‐κB–dependent production of IL‐1β and TNF‐α, both of which are known to disrupt endothelial junctional complexes [73]. Moreover, MP‐associated stress can activate the NLRP3 inflammasome, with caspase‐1–dependent IL‐1β maturation triggering RhoA/ROCK‐mediated cytoskeletal contraction and rapid internalization of tight‐junction proteins [74]. Increased intracellular Ca^2^^+^ and stress fiber formation introduce additional mechanical tension, further weakening junctional stability. Notably, recent evidence shows that MP–LPS complexes induce far greater downregulation of claudin‐5 and occludin than either exposure alone, underscoring their synergistic neurovascular toxicity [23].

Together, these pathways position ROS/NF‐κB–centered tight‐junction disruption—further amplified by LPS‐adsorbed MPs and inflammasome activation—as a central mechanistic bridge linking MP exposure to BBB breakdown, neuroinflammation, and potentially heightened stroke susceptibility. Within this mechanistic framework, MP‐induced injury to BMECs is not triggered by a single process but arises from multiple synergistic events, including mitochondrial dysfunction, excessive ROS accumulation, activation of NF‐κB/MAPK/NLRP3 signaling, and sustained downregulation of tight‐junction proteins, collectively disrupting barrier homeostasis. This injury exhibits clear dose dependence and polymer specificity, with BMEC apoptosis typically emerging 12–24 h after exposure and accompanied by increased barrier permeability. Although many experimental studies employ doses higher than environmentally relevant levels, low‐dose, long‐term exposure models still reveal detectable BBB impairment, underscoring the potential neurovascular risks of MPs in human populations.

Overall, MPs disrupt the BBB through multiple synergistic mechanisms, centered on ROS/NF‐κB–mediated tight‐junction impairment and further amplified by LPS adsorption and inflammasome activation, ultimately causing BMEC apoptosis, increased barrier permeability, and heightened susceptibility to neuroinflammation. Notably, BBB dysfunction is observed even under low, environmentally relevant long‐term exposures, suggesting that MPs may pose a significant neurovascular risk to human populations.

### 4.2 Neuroinflammation and Microglial Activation

Once MPs traverse the BBB and enter brain parenchyma, they primarily interact with microglia—the CNS's resident innate immune sentinels [21]. Under homeostatic conditions, microglia maintain a surveillant state essential for synaptic remodeling and debris clearance [75]. However, in the neurotoxic milieu induced by MP exposure, microglia rapidly transition into an activated, predominantly M1‐like pro‐inflammatory phenotype. These M1 microglia secrete high levels of TNF‐α, IL‐6, IL‐1β, IL‐18, nitric oxide, and ROS, collectively damaging axons, synapses, and myelin structures [76] (Figure 4). Multiple rodent studies have shown that PS MP exposure markedly increases Iba‐1–positive microglia in the hippocampus and cortex, accompanied by hypertrophic morphology and elevated pro‐inflammatory marker expression [77, 78, 79]. These findings suggest that MPs may function as “danger‐associated molecular patterns”(DAMPs), aberrantly activate microglia and initiate a robust neuroinflammatory cascade. Sustained microglial activation not only amplifies neuronal injury but also establishes a self‐perpetuating cycle of chronic neuroinflammation—an important pathological bridge linking MP exposure to heightened stroke susceptibility and broader neurodegenerative processes.

Moreover, pro‐inflammatory cytokines released by M1‐polarized microglia exert not only local neurotoxic effects but also sustain a self‐amplifying inflammatory loop, driving a persistent neuroinflammatory state. These mediators act on astrocytes, neurons, and brain microvascular endothelial cells, disrupting neural homeostasis and circuit function [80, 81]. TNF‐α and IL‐6 impair synaptic plasticity and increase BBB permeability [82], whereas IL‐18 is critically involved in ischemic stroke pathogenesis and secondary injury [83]. Prolonged M1 activation further suppresses the transition toward the M2 reparative phenotype, depriving injured tissue of essential regenerative cues. Under chronic or repeated exposure, this imbalance may progress to neuroinflammatory priming—characterized by low‐grade yet persistent inflammation—which increases susceptibility to neurodegeneration, cognitive decline, and stroke [84, 85, 86]. Thus, MP‐induced M1 polarization and sustained cytokine release act as key amplifiers of neurovascular injury and cerebrovascular vulnerability.

Collectively, MPs that reach the brain aberrantly activate microglia into a sustained M1‐like pro‐inflammatory state, driving excessive cytokine and ROS production that damages neurons, disrupts BBB integrity, and establishes a self‐amplifying neuroinflammatory loop. This chronic microglial priming markedly increases neurovascular vulnerability, providing a key pathological link between MP exposure, stroke susceptibility, and broader neurodegenerative risk.

### 4.3 Cerebral Microvascular Injury and Impaired Brain Perfusion

The cerebral microvasculature, the smallest functional unit supporting CNS perfusion, is essential for sustaining neuronal metabolism, clearing metabolic waste, and maintaining neural network function. After entering the brain through systemic circulation, MPs can directly interact with cerebral microvascular endothelial cells, inducing both functional impairment and structural injury [63, 64]. MP exposure has been shown to markedly reduce endothelial nitric oxide (NO) production by downregulating or inhibiting endothelial nitric oxide synthase (eNOS) [87, 88]. Because eNOS is central to vasodilation and vascular homeostasis, its dysfunction leads to dysregulated vascular tone, focal microvascular constriction, and impaired cerebral perfusion [89]. In parallel, MPs promote endothelial activation by upregulating adhesion molecules such as ICAM‐1 and VCAM‐1, thereby enhancing leukocyte adhesion and initiating inflammatory signaling [90]. This endothelial inflammation increases microvascular permeability and destabilizes local hemodynamics. With sustained exposure, chronic low‐grade inflammation contributes to vascular basement membrane thickening and luminal narrowing—pathological features strongly associated with reduced cerebral perfusion and heightened neurovascular vulnerability [91].

On a functional level, MP exposure markedly disrupts the autoregulation of cerebral blood vessels. Under physiological conditions, the cerebral microvasculature tightly adjusts vascular tone to match regional metabolic demand and maintain stable perfusion [92]. Emerging evidence indicates that MPs impair Ca^2^^+^ channel function and disturb the contractile activity of cerebral vascular smooth muscle cells, leading to abnormal responses to vasoactive stimuli such as acetylcholine (Ach) and norepinephrine (NE) [93, 94]. These alterations blunt or exaggerate vasomotor responses, resulting in perfusion instability. Under inflammatory conditions, cerebral vessels may develop excessive vasoconstriction or local hypoperfusion, increasing susceptibility to focal ischemia—particularly in metabolically active regions such as the hippocampus and cortex [94]. When combined with additional stressors, including acute psychological stress or systemic hypertension, such microvascular dysfunction may act as a trigger for ischemic stroke. Furthermore, impaired microcirculation compromises BBB repair, metabolic waste clearance, and neural stem cell niche regeneration, thereby diminishing CNS resilience and recovery capacity [95, 96, 97]. Collectively, MPs disrupt endothelial function, upregulate inflammatory adhesion molecules, and impair hemodynamic regulation, culminating in cerebral microvascular injury and perfusion deficits—key upstream events in the development of cerebrovascular diseases, including stroke.

In summary, MP exposure may contribute to cerebrovascular injury through a series of interconnected biological processes. First, MPs can disrupt the intestinal epithelial barrier, leading to gut microbiota dysbiosis and increased intestinal permeability, which together drive a persistent state of systemic inflammation [98, 99]. This systemic inflammatory milieu can further compromise the structural integrity and permeability of the BBB, facilitating the entry of external particles, pro‐inflammatory mediators, and oxidative stress signals into the central nervous system. Notably, a study published in The *New England Journal of Medicine* analyzed carotid endarterectomy specimens from 304 patients and detected PE in more than 58% of plaques. Over nearly three years of follow‐up, patients with MNP‐positive plaques exhibited a 353% higher risk of myocardial infarction, stroke, or all‐cause mortality compared with those without detectable MNPs [91], suggesting a potential association between MP exposure, vascular inflammation, and plaque instability. In parallel, MP‐related signals can activate microglia and promote their polarization toward a pro‐inflammatory phenotype, thereby amplifying neuroinflammatory responses within the central nervous system [21, 100]. Moreover, MPs may directly impair cerebrovascular endothelial function, reducing vasodilatory capacity and disturbing cerebral perfusion homeostasis [101]. Together, these multilayered pathological alterations form a sequential mechanistic cascade—from intestinal barrier disruption and systemic inflammation to BBB impairment, neuroinflammation, and cerebrovascular dysfunction—which provides a biological basis for the potential link between MP exposure, cerebrovascular injury, and increased stroke susceptibility.

Overall, MPs impair cerebral microvascular function by reducing eNOS‐derived nitric oxide, promoting endothelial inflammation, and destabilizing hemodynamic regulation, ultimately leading to perfusion deficits and heightened vulnerability to ischemia. Together with systemic inflammation and BBB impairment, these vascular disturbances form a mechanistic cascade linking MP exposure to cerebrovascular injury and increased stroke susceptibility.

## 5 Potential Mechanisms Linking MPs and Stroke

### 5.1 Formation of a Pro‐Stroke Inflammatory Environment

**FIGURE 5:**
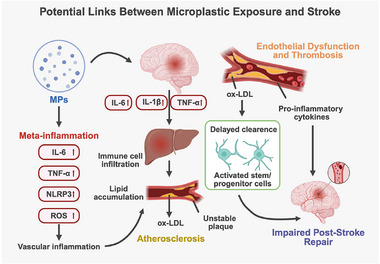
Potential pathways through which MP exposure may modulate stroke susceptibility and post‐stroke recovery. Chronic MP exposure may be associated with a low‐grade pro‐inflammatory vascular milieu, potentially involving oxidative stress‐related signaling and inflammasome‐associated pathways (e.g., NLRP3), which could influence endothelial function and pro‐thrombotic states under specific conditions. MPs may also affect atherosclerotic processes via alterations in lipid handling and plaque stability in a context‐dependent manner. Following stroke, sustained neuroinflammatory signaling and microglial activation may interfere with neural stem/progenitor cell (NSPC) activity, neurogenesis, and angiogenesis, thereby limiting functional recovery. The pathways depicted represent one possible integrative framework, and additional or alternative mechanisms may contribute to MP‐associated neurovascular effects. Created with BioRender.

Chronic exposure to MPs, as a pervasive environmental contaminant, has been increasingly linked to sustained low‐grade systemic inflammation—referred to as metabolic inflammation or meta‐inflammation (Figure 5)—a recognized risk enhancer for cerebrovascular disease, including stroke [102]. In rodent models, chronic exposure typically refers to continuous administration for ≥4‐12 weeks, primarily via oral ingestion, with doses ranging from environmentally relevant levels (approximately 10–100 µg/kg/day) to commonly used experimental doses (1–10 mg/kg/day) [7, 24, 103]. Unlike acute inflammation, meta‐inflammation represents a chronic, subtle inflammatory state driven by cumulative environmental and metabolic stressors, often remaining subclinical while causing widespread physiological disruption [104, 105, 106]. Experimental evidence demonstrates that MP exposure induces persistent elevations of circulating IL‐6, TNF‐α, and IL‐1β, accompanied by immune cell activation in organs such as the spleen, liver, and brain [107, 108, 109, 110]. This chronic inflammatory milieu promotes endothelial dysfunction, destabilizes atherosclerotic plaques, enhances coagulation, and impairs cerebrovascular autoregulation—collectively heightening ischemic stroke susceptibility [91, 111]. In parallel, long‐term inflammation can activate the HPA axis and disrupt neuroendocrine homeostasis, further predisposing the CNS to cerebrovascular events [112].

At the molecular level, the NLRP3 inflammasome has emerged as a central mediator linking MP exposure to heightened stroke susceptibility (Figure 5). As a key intracellular inflammatory sensor, NLRP3 is activated by oxidative stress, ionic imbalance, and mitochondrial dysfunction, leading to caspase‐1 activation and the maturation of IL‐1β and IL‐18 [113]. Growing evidence shows that MPs markedly induce NLRP3 activation by promoting ROS accumulation and mitochondrial impairment, thereby amplifying pro‐inflammatory immune phenotypes [8, 114, 115, 116]. This cascade aggravates vascular inflammation, impairs endothelial function, and promotes platelet activation and microthrombus formation—critical events that compromise cerebral perfusion. Under chronic inflammatory states, NLRP3 can also become “primed”, enabling minor physiological perturbations, such as transient ischemia or blood pressure fluctuations, to trigger disproportionately strong inflammatory responses and accelerate stroke onset [117, 118, 119]. Thus, MP‐induced low‐grade inflammation, driven by NLRP3 and ROS‐dependent pathways, establishes a prothrombotic, high‐risk microenvironment that reduces CNS resilience to acute insults and increases vulnerability to cerebrovascular disease.

It is important to note that activation of the NLRP3 inflammasome represents only one of several potential mechanisms by which MPs may induce neurovascular injury [120]. Increasing evidence suggests that systemic low‐grade inflammation, metabolic reprogramming of cerebrovascular endothelial cells, amplification of gut–brain axis signaling, and protein‐corona–mediated cellular interactions may independently contribute to neurovascular toxicity even in the absence of NLRP3 activation [121]. Moreover, several in vitro studies have reported no significant activation of NLRP3, indicating that involvement of this pathway is dose‐dependent, polymer‐dependent, and varies across cell types. These findings underscore that NLRP3 activation is not a universal mechanism and should be interpreted within a broader biological context.

Consequently, chronic MP exposure induces persistent low‐grade systemic inflammation and NLRP3–ROS–driven immune activation, creating a prothrombotic and cerebrovascular‐vulnerable state that heightens susceptibility to ischemic stroke. However, NLRP3 activation is not universal; its involvement varies with dose, polymer type, and cell context, indicating that MPs may exert neurovascular toxicity through multiple parallel pathways.

### 5.2 Endothelial Dysfunction and Prothrombotic Tendency

Vascular endothelial cells are essential for maintaining vascular integrity, regulating vasomotor tone, and sustaining hemodynamic stability, and their dysfunction is an early hallmark of vascular disorders, including atherosclerosis and stroke [122]. Emerging evidence indicates that MP exposure directly impairs endothelial cells, inducing morphological abnormalities, apoptosis, and increased permeability, ultimately weakening endothelial barrier function [116, 123, 124]. This injury is frequently accompanied by reduced eNOS expression and diminished NO bioavailability, resulting in impaired vasodilation, vasospasm, and disturbed cerebral perfusion [94]. In parallel, MPs activate pro‐inflammatory signaling pathways such as NF‐κB and MAPK, driving endothelial release of cytokines including IL‐6, IL‐8, and MCP‐1 [114, 125]. This inflammatory milieu promotes vascular adhesion, further amplified by MP‐induced upregulation of adhesion molecules (ICAM‐1, VCAM‐1) and Tissue Factor (TF) (Figure 5), thereby enhancing leukocyte and platelet adhesion, activation, and aggregation—key steps in thrombogenesis. Long‐term MP exposure in animal models has also been associated with arterial wall thickening, subendothelial immune cell infiltration, and luminal narrowing, collectively indicating that MPs can induce systemic vascular injury and elevate the risk of cerebral infarction [94, 126].

Further mechanistic studies indicate that MP‐induced platelet activation is a key driver of their prothrombotic effects. Once in circulation, MPs may act as foreign surfaces that directly interact with platelet membrane receptors or indirectly bind plasma proteins such as fibrinogen, thereby facilitating receptor engagement. These interactions trigger platelet activation, characterized by shape change, degranulation, and the release of procoagulant mediators such as ADP, collectively promoting aggregation and accelerating the coagulation cascade [127, 128, 129].MP exposure has also been associated with increased circulating levels of fibrinogen and prothrombin, further enhancing hypercoagulability [130, 131]. Concurrent upregulation of Tissue Factor (TF) persistently activates the extrinsic coagulation pathway, expediting thrombus initiation and stabilization. Within the cerebrovascular system, this prothrombotic milieu—when combined with endothelial injury, focal hypoperfusion, or plaque rupture—can readily precipitate acute cerebral infarction [132]. Importantly, MP‐induced endothelial dysfunction and thrombogenesis extend beyond localized vascular effects, contributing to a systemic “pro‐stroke state” reinforced by chronic inflammation, oxidative stress, and immune dysregulation. Thus, MPs may act not merely as distal environmental contaminants but as active instigators of a “pro‐stroke triad” comprising vascular inflammation, endothelial injury, and thrombogenesis, collectively amplifying cerebrovascular vulnerability [111].

Taken together, MP exposure promotes a “pro‐stroke triad” of endothelial dysfunction, vascular inflammation, and thrombogenesis by impairing eNOS‐NO signaling, activating NF‐κB/MAPK pathways, upregulating adhesion molecules and Tissue Factor, and directly triggering platelet activation. These synergistic effects create a systemic prothrombotic and vasculature‐vulnerable state that markedly increases the risk of cerebral infarction.

### 5.3 Progression of Atherosclerosis

Atherosclerosis— the principal pathological basis of ischemic stroke—arises from a complex interplay of lipid accumulation, chronic inflammation, endothelial dysfunction, and vascular remodeling [133]. Emerging evidence indicates that MPs, as persistent environmental toxicants, can disrupt lipid metabolic homeostasis at multiple regulatory levels, thereby accelerating atherogenesis [134].MP‐induced gut microbiota alterations and bile acid dysregulation impair cholesterol absorption and excretion, fostering a pro‐atherogenic metabolic state. Animal studies have shown that exposure to polystyrene or polyethylene MPs significantly increases plasma total cholesterol and LDL‐C while reducing HDL‐C, reflecting a lipid profile conducive to plaque development [7]. Additionally, MPs can directly influence hepatic lipid metabolism. Experimental evidence demonstrates that MP exposure activates lipogenic pathways in hepatocytes, enhances lipid synthesis, and increases circulating atherogenic lipoprotein particles. These lipids accumulate within the vascular endothelium, promoting plaque formation and vascular wall thickening [135, 136, 137].

On the other hand, the inflammatory milieu and oxidative stress induced by MP exposure markedly enhance the formation of lipid oxidation products, particularly oxidized LDL (ox‐LDL), a critical driver of atherogenesis. Ox‐LDL promotes macrophage‐to‐foam‐cell transformation and activates pro‐inflammatory pathways such as TLR4/NF‐κB, thereby amplifying vascular inflammation. This inflammatory cascade stimulates smooth muscle cell migration and proliferation, collagen deposition, and fibrous cap thickening, collectively accelerating plaque progression [138, 139]. Experimental studies demonstrate that MP‐exposed animals develop significantly larger lipid‐rich aortic plaques with greater inflammatory cell infiltration and vascular wall thickening compared to controls [107]. More importantly, in major cerebral arteries such as the carotid and middle cerebral arteries, MP‐associated plaque accumulation results in luminal stenosis, vessel wall fragility, and plaque instability—hallmarks that sharply increase the risk of plaque rupture and thromboembolic events [140]. These vulnerabilities are further exacerbated in individuals with comorbidities such as hypertension or diabetes [110]. Thus, by disrupting lipid metabolism, intensifying vascular inflammation, and promoting atheromatous plaque progression, MP exposure emerges as an important environmental contributor to cerebral atherosclerosis and ischemic stroke.

Overall, MPs accelerate atherosclerosis—the key pathological substrate of ischemic stroke—by disrupting lipid metabolism, elevating LDL‐C/ox‐LDL, and amplifying vascular inflammation, thereby promoting foam‐cell formation, plaque growth, and instability. These combined metabolic and inflammatory effects narrow cerebral arteries and heighten the risk of plaque rupture and thromboembolic stroke.

### 5.4 Impeded Post‐Stroke Repair Mechanisms

Following a stroke, effective tissue repair and functional recovery depend heavily on the activation and neurogenic potential of neural stem/progenitor cells (NSPCs) [141, 142]. However, MP‐induced chronic neuroinflammation and oxidative stress markedly compromise this regenerative microenvironment. Experimental evidence shows that PS MPs suppress NSPC proliferation and differentiation in key neurogenic niches—such as the hippocampal dentate gyrus and subventricular zone (SVZ)—mainly through sustained elevation of IL‐6 and TNF‐α driven by TLR4/NF‐κB signaling and NLRP3 inflammasome activation [12]. This pro‐inflammatory state is further amplified by excessive ROS accumulation, mitochondrial dysfunction, and apoptotic signaling, collectively impairing the post‐stroke neural repair cascade. In parallel, MP‐induced reactive gliosis—characterized by persistent M1 microglial activation and astrocytic reactivity—releases neurotoxic mediators including IL‐1β, NO, and reactive enzymes, thereby inhibiting axonal regeneration and synaptic remodeling [47]. These converging processes disrupt neuroplasticity and glial scaffold formation, ultimately undermining the efficacy of endogenous neurorestorative responses.

Moreover, MP exposure may impede the timely clearance of necrotic tissue and inflammatory debris after stroke, thereby prolonging neuroinflammation and exacerbating secondary injury. Efficient removal of apoptotic and necrotic cells—known as immunological clearance—is essential for integrating newly generated cells and promoting tissue reconstruction [143]. Under physiological conditions, microglia and infiltrating monocytes undergo a subacute transition toward the anti‐inflammatory M2 phenotype, secreting reparative cytokines such as IL‐10 and TGF‐β and facilitating debris phagocytosis [144]. Chronic MP exposure disrupts this phenotypic switch, sustaining a dominance of pro‐inflammatory M1‐polarized immune cells. Consequently, the infarcted region persists in a high‐ROS, pro‐inflammatory microenvironment [145, 146], delaying inflammation resolution. This impairs NSPC migration, disrupts angiogenesis, and interferes with glial scar remodeling, ultimately compromising neural repair. As a result, post‐stroke recovery becomes delayed or incomplete, often manifesting as cognitive decline, motor dysfunction, and heightened seizure susceptibility. Thus, MPs may not only promote stroke onset but also aggravate long‐term disability by impairing endogenous regenerative mechanisms.

Consequently, MPs impede post‐stroke recovery by sustaining chronic neuroinflammation and oxidative stress, which suppress neural stem cell proliferation, disrupt neurogenic niche function, and inhibit axonal and synaptic repair. By blocking the essential shift from M1 to M2 phenotypes and delaying debris clearance, MPs prolong secondary injury and ultimately undermine neural regeneration and functional recovery.

## 6 Evidence, Mechanisms, and Challenges of MP‐Related Cerebrovascular Risk

### 6.1 Human Epidemiological Evidence and Inferences

**FIGURE 6:**
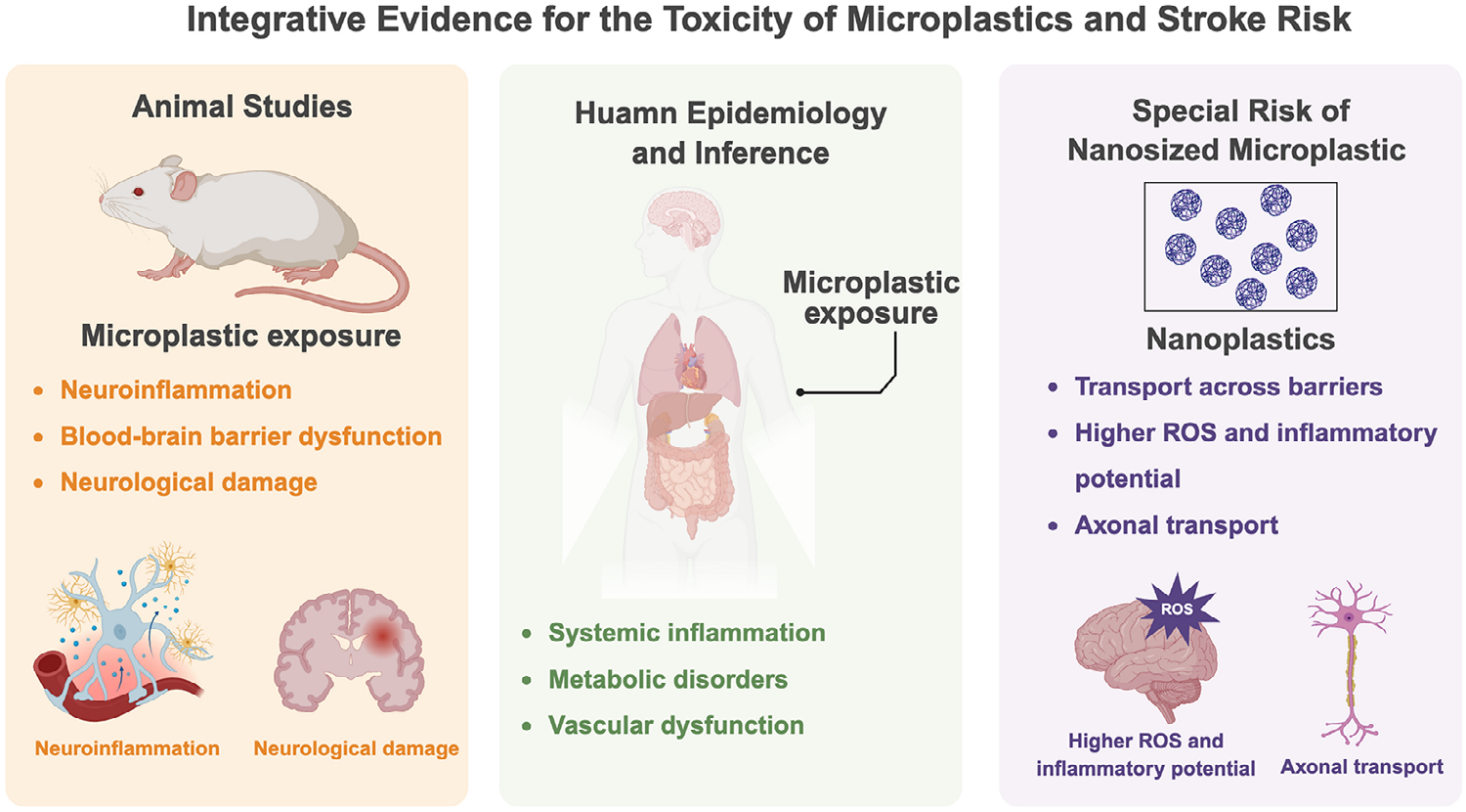
Conceptual illustration of associations between MP exposure and cerebrovascular vulnerability. Chronic MP exposure may, under specific conditions, be associated with neuroinflammatory signaling, microglial activation, alterations in BBB integrity, and cognitive changes. In ischemic contexts, MP exposure may also coincide with increased infarct severity and delayed functional recovery, with effects that are highly context dependent. Population‐level observations suggest associations between MP‐related exposure indicators and systemic inflammation, vascular dysfunction, or metabolic disturbances, which are recognized modifiers of cerebrovascular risk. Smaller particles, including nanoplastics, may exhibit greater biological accessibility, potentially involving neuronal uptake routes such as axonal transport and subsequent subcellular stress responses. Overall, this figure integrates associative signals across different lines of evidence to conceptually depict possible links between MP exposure and neurovascular outcomes. Created with BioRender.

Although animal studies have outlined the neurotoxic and vascular effects of MPs, direct epidemiological evidence linking MP exposure to stroke risk in humans remains limited (Figure 6). Existing human data are largely indirect, including associations between higher MP exposure and elevated inflammatory biomarkers such as CRP [147], increased carotid intima‐media thickness [91], and exacerbated dyslipidemia or insulin resistance driven by gut dysbiosis and intestinal barrier impairment [8, 148]. These inflammatory, metabolic, and vascular abnormalities—all established risk factors for ischemic stroke—suggest the emergence of a potential “ high‐risk phenotype.” However, causality cannot be established due to the absence of large, well‐designed prospective cohort studies.

Multiple translational limitations further complicate the interpretation of the current evidence. Rodent studies often employ relatively high or tightly controlled exposure levels, and species‐specific differences in particle uptake, immune responses, and intestinal or BBB physiology may lead to an overestimation of human neurovascular risk. Notably, rodents exhibit higher basal metabolic rates and faster intestinal transit, which under experimental exposure conditions may substantially enhance particle uptake efficiency, systemic distribution, and the magnitude of inflammatory responses [149, 150]. In addition, fundamental interspecies differences in BBB structure and transport properties—including the organization of endothelial tight junctions, transporter expression profiles, and immune‐endothelial interaction patterns—may facilitate the translocation of micro‐ or nanoplastics across the barrier and amplify neuroinflammatory effects in rodents, particularly at exposure levels exceeding those encountered in real‐world human environments [48, 50, 151]. Consequently, existing rodent studies primarily support hazard identification rather than quantitative assessment of human neurovascular risk, and their translational relevance should be interpreted cautiously considering exposure levels and species differences.

Meanwhile, although in vitro models provide important mechanistic insights, they substantially oversimplify the neurovascular microenvironment, failing to capture the complexity of multicellular interactions or to replicate the chronic low‐dose exposure scenarios typical of human populations. Moreover, standardized biomonitoring protocols for detecting MPs of different polymer types and particle sizes in human biospecimens are currently lacking [152, 153, 154], further reducing the reliability and comparability of exposure assessments. Finally, most existing epidemiological studies are cross‐sectional or limited in sample size, with insufficient adjustment for lifestyle factors, co‐exposures, and underlying metabolic conditions, thereby constraining causal inference and the generalizability of the findings.

In addition, recent monitoring data indicate that the measured levels of MPs in daily human exposure sources fall within a defined range: approximately 1–10^3^ particles/L in drinking water, 10^2^–10^5^ particles/L in bottled water, 10^2^–10^6^ particles/kg in seafood, and 10^2^–10^4^ particles/kg in table salt, while airborne deposition may contribute 10^1^–10^4^ particles/m^3^ to inhalation exposure [6, 103]. When compared with these real‐world exposure levels, the doses used in many animal studies are substantially higher and often far exceed the amounts humans encounter environmentally, potentially leading to an overestimation of toxicity. Based on current evidence, an “ environmentally relevant dose” should correspond to the above measured exposure ranges and generally translate to approximately µg/kg–mg/kg body weight per day, rather than the traditionally adopted high‐dose regimens of 10–100 mg/kg commonly used in toxicological studies. Clarifying this dose discrepancy is essential for accurately interpreting animal research findings, improving the environmental relevance of experimental designs, and reliably assessing the potential health risks of MP exposure.

Importantly, MPs themselves exhibit substantial physicochemical heterogeneity. Variations in polymer composition, particle size distribution, morphology (e.g., spherical, fibrous, fragment‐like forms), and environmental aging processes such as photooxidation or microbial degradation can profoundly influence their biological interactions and toxicological profiles. These factors affect particle deposition, biodistribution, cellular uptake, and the intensity of inflammatory or oxidative stress responses, contributing to inconsistent findings across experimental and epidemiological studies. Moreover, the lack of standardized exposure metrics—such as dose units, size‐class definitions, and analytical detection sensitivity—further complicates risk assessment and remains a major limitation in evaluating potential health hazards.

In addition, multiple confounding factors—including lifestyle, genetic susceptibility, and co‐exposure to other environmental pollutants—may influence cerebrovascular outcomes through independent or interactive pathways, substantially increasing the difficulty of isolating the independent health effects of MP exposure and limiting the interpretability of existing epidemiological evidence. From the perspective of real‐world environmental exposure and population‐based research, it becomes evident that these confounders, together with methodological limitations, jointly constrain causal inference regarding MP‐related cerebrovascular effects at multiple levels.

In real environmental settings, MPs rarely occur as a single pollutant but instead constitute an integral component of complex exposure mixtures, coexisting extensively with particulate matter (PM_2_._5_/PM_10_), heavy metals, and persistent organic pollutants [155, 156]. Under such co‐exposure scenarios, different pollutants not only share overlapping sources but also converge on several key biological pathways, including endothelial dysfunction, amplification of oxidative stress, activation of chronic low‐grade inflammation, and pro‐thrombotic and vascular remodeling processes [157]. Consequently, in the absence of comprehensive multi‐pollutant adjustment, adverse cerebrovascular outcomes observed in population studies are difficult to attribute specifically to MP exposure. Meanwhile, lifestyle‐related factors (e.g., dietary patterns, smoking and alcohol consumption, and physical activity), socioeconomic status, and genetic backgrounds influencing immune and metabolic regulation often correlate simultaneously with both MP exposure levels and cerebrovascular outcomes, yet are insufficiently measured or systematically adjusted for in existing studies, further increasing the risk of residual confounding.

From a study design and statistical analysis standpoint, current population‐based investigations of MP exposure and cerebrovascular health remain largely exploratory. On the one hand, most studies adopt cross‐sectional or retrospective designs and rely on single‐time‐point or short‐term exposure assessments, limiting their ability to capture long‐term and cumulative exposure patterns and to establish temporal relationships between exposure and disease onset [158, 159]. On the other hand, most analyses are based on single‐pollutant models that fail to adequately account for correlations, collinearity, and potential interactions among multiple exposure factors, thereby increasing the likelihood of biased effect estimates [160, 161]. Although some studies have reported associations between MPs and inflammatory biomarkers or vascular structural alterations, insufficient control for key confounders such as air pollution, heavy metal burden, or metabolic status renders these findings subject to considerable uncertainty. Overall, the existing evidence more often reflects the co‐occurrence of exposure‐related signals rather than the independent causal contribution of MPs to cerebrovascular disease.

Therefore, to more effectively delineate the independent health effects of MP exposure, future research requires systematic methodological advancement. On the one hand, multi‐pollutant regression models and mixed‐exposure analytical frameworks should be adopted to simultaneously evaluate the effects of MPs alongside other relevant environmental pollutants, thereby reducing collinearity and confounding bias [162, 163]. On the other hand, exposome‐wide association studies (ExWAS) offer a powerful approach for systematically identifying exposure factors and exposure patterns most strongly associated with cerebrovascular outcomes in high‐dimensional exposure contexts [164, 165]. Further integration of genetic information through stratified analyses or Mendelian randomization approaches may help mitigate confounding from lifestyle and environmental factors and reduce the risk of reverse causation [166, 167, 168].

Given the multifactorial etiology of stroke, future research should also prioritize the establishment of large‐scale longitudinal population cohorts alongside the development of standardized human biomonitoring frameworks for MPs to improve the reliability and comparability of exposure assessment. Building upon these foundations, integrating exposomics with multi‐omics platforms and AI‐based modeling strategies will be essential for disentangling complex exposure–response relationships. Moreover, targeted investigations in high‐risk populations—such as older adults, individuals with metabolic syndrome, and residents of highly urbanized or industrial regions—may help identify susceptibility windows and support more precise cerebrovascular risk assessment. Collectively, these efforts are critical for generating robust human evidence, clarifying the cerebrovascular consequences of chronic MP exposure, and advancing the field from hazard identification toward evidence‐based risk assessment.

Overall, human epidemiological evidence linking MP exposure to cerebrovascular risk remains limited and largely indirect. Discrepancies between experimental and environmentally relevant exposures, along with physicochemical heterogeneity, species differences, and pervasive confounding, constrain causal inference. As such, existing findings are best viewed as hypothesis‐generating, underscoring the need for standardized exposure assessment and robust longitudinal studies.

### 6.2 The Unique Risks Posed by Nanoplastics

Compared with their micro‐sized counterparts, nanoplastics have drawn increasing scientific concern owing to their nanoscale dimensions, high specific surface area, and markedly enhanced biological reactivity [169, 170] (Figure 6). Their small size confers an exceptional ability to traverse biological barriers, enabling efficient penetration of the gastrointestinal epithelium, BBB, and even the placental interface [98, 171]. Accumulating in vivo and in vitro evidence shows that NPs are readily internalized by epithelial and endothelial cells via transcellular routes—including endocytosis and exocytosis—and subsequently trafficked to intracellular organelles, where they induce mitochondrial dysfunction, lysosomal destabilization, and endoplasmic reticulum stress [172]. These neurotoxic properties are particularly striking within the CNS. Following oral or intravenous exposure, NPs can reach the brain rapidly and preferentially accumulate in metabolically active and functionally critical regions such as the hippocampus, striatum, and thalamus, indicating high neurobioavailability [14, 173]. At the molecular level, NPs readily acquire a protein corona in biological fluids, facilitating interactions with plasma proteins, membrane receptors, and nucleic acids, thereby reshaping their biodistribution, cellular uptake kinetics, and toxicity signatures [174]. Notably, when compared to larger MPs of identical polymer composition, NPs consistently display higher surface reactivity, resulting in amplified ROS generation, heightened pro‐inflammatory cytokine release, and stronger activation of apoptotic pathways [175].

Of particular concern, emerging evidence suggests that NPs may access deep brain structures through both axonal transport and regulated transcellular pathways across the blood–brain barrier (BBB). Similar to certain neurotropic viruses (e.g., HSV, rabies) and neurotoxins, NPs can be internalized at peripheral nerve terminals and conveyed retrogradely along microtubule‐based axonal pathways to central regions such as the brainstem, midbrain, and limbic system [62, 176, 177, 178]. Co‐culture studies further indicate that NPs can traverse synaptic junctions, enter axons, and disrupt synaptic protein homeostasis and neurotransmitter signaling [179, 180], thereby potentially contributing to cognitive decline, emotional dysregulation, and impaired motor recovery after stroke.

Concurrently, NPs can cross the BBB through several endocytosis‐mediated transcellular routes. Clathrin‐mediated endocytosis (CME) represents a major uptake mechanism for particles <200 nm, and pharmacological inhibition with chlorpromazine markedly reduces endothelial internalization [101, 181, 182, 183]. Caveolin‐mediated endocytosis also contributes to NP uptake, particularly for smaller or surface‐modified particles, while inflammatory conditions enhance macropinocytosis, increasing non‐specific NP engulfment. Following endocytosis, NPs may undergo transcytosis through early and late endosomal trafficking pathways, regulated by small GTPases such as Rab11 and Rab7, enabling apical‐to‐basolateral movement into brain parenchyma.

Beyond barrier penetration, NPs may accumulate within cerebral vessel walls or the ventricular system, eliciting chronic microinflammation, oxidative stress, and focal perfusion deficits—factors known to heighten susceptibility to ischemic injury [11, 184]. Due to their ultrafine size and the current limitations of analytical platforms, NPs frequently evade detection, leaving major gaps in understanding their biodistribution, clearance kinetics, and neurovascular toxicity [185, 186]. Addressing these challenges will require high‐sensitivity detection technologies and multi‐scale toxicological models capable of resolving NP–CNS interactions with precision.

The proposed mechanism of axonal transport of nanoplastics is currently supported only by rodent models and primary neuron culture systems, with no direct evidence in humans. Most existing findings are derived from high‐dose exposures or tightly controlled laboratory conditions, which may not accurately reflect physiological scenarios under environmentally relevant exposure levels. Therefore, this mechanism should be regarded as a hypothesis with heuristic value rather than an established biological process. Future studies incorporating human biomonitoring, advanced organoid models, and multimodal imaging technologies will be essential to determine the relevance of this mechanism to cerebrovascular pathology in real‐world human populations.

Collectively, nanoplastics pose distinct neurovascular risks due to their exceptional ability to cross biological barriers—including the gut epithelium, BBB, and even neural pathways—where they accumulate in sensitive brain regions and trigger oxidative stress, inflammation, and organelle dysfunction. Although current evidence largely stems from high‐dose animal and in vitro studies, these findings highlight the need for advanced human‐relevant models to clarify the real‐world cerebrovascular impact of nanoplastic exposure.

### 6.3 Challenges of Multi‐Omics Integration, Artificial Intelligence, and Methodological Standardization in MP Research

Although the integration of multi‐omics and AI technologies holds considerable promise for elucidating MP‐related health effects, exposure characteristics, and potential mechanisms, their practical application in current environmental health research remains constrained by several critical technical bottlenecks [187, 188]. First, substantial differences in detection sensitivity, dynamic range, and data structure across omics platforms readily introduce batch effects and platform‐specific biases, thereby undermining cross‐study comparability [189, 190]. At the same time, multi‐omics datasets typically exhibit a “high‐dimensional–small‐sample” structure, which is particularly pronounced in environmental exposure research and markedly increases statistical instability and the risk of overfitting [191, 192]. In the absence of unified cross‐platform data processing and quality‐control pipelines, as well as sufficiently large sample sizes, the reproducibility and external validity of multi‐omics findings remain limited.

AI‐based approaches likewise show broad potential in MP exposure assessment, risk prediction, and mechanistic inference, but their application is currently hampered by the scarcity of high‐quality training datasets and the lack of reliable real‐world exposure labels. The pronounced physicochemical heterogeneity of MPs—including variations in particle size, morphology, and polymer composition—may introduce systematic bias during model development [187, 193]. Moreover, the limited interpretability of deep learning algorithms complicates the translation of model outputs into biologically testable mechanistic hypotheses [190, 194]. Collectively, these issues restrict the standardized and generalizable use of AI tools in MP health research.

The lack of standardization in MP biomonitoring represents another major bottleneck for exposure assessment and epidemiological comparison. Internationally recognized certified reference materials and unified calibration systems are still unavailable, making it difficult to directly compare results across studies that differ in particle size ranges, polymer classifications, and reporting units [195, 196]. In addition, substantial methodological variability exists across laboratories in key steps such as sample collection, digestion, filtration and enrichment, particle isolation, and spectroscopic identification (e.g., FTIR, Raman), further amplifying cross‐study and cross‐platform inconsistency. Current analytical instruments also have limited resolution for particle size and polymer identification, particularly for MPs smaller than 1 µm, which may lead to systematic underestimation of true exposure levels. Meanwhile, sample collection and processing are susceptible to environmental contamination, potentially generating false‐positive or false‐negative signals. Together, these technical limitations indicate that MP exposure data used in current epidemiological studies should be interpreted cautiously with respect to their reliability and comparability.

To advance the field from exploratory research toward mechanistic elucidation and quantitative health risk assessment, a phased and operational roadmap is required. First, priority should be given to standardizing sample processing, MP detection, and omics data acquisition workflows, accompanied by the establishment of cross‐laboratory quality‐control and reference systems to reduce bias arising from technical heterogeneity [25, 197]. Second, high‐quality, multidimensional omics databases should be constructed based on standardized protocols to provide a robust foundation for downstream analyses [198]. At the analytical level, staged model development and validation strategies are needed, with clear separation between exploratory analyses, model training, and independent validation, complemented by cross‐validation, multi‐center datasets, and external validation to assess model stability and generalizability. For high‐dimensional data, feature selection, dimensionality reduction, and sparse modeling approaches should be prioritized to avoid indiscriminate reliance on overly complex model architectures. Ultimately, these standardized and model‐integrated strategies should be progressively implemented in prospective population studies, enabling more accurate evaluation of health risks associated with MP exposure and providing reliable, interpretable evidence to inform scientific decision‐making in environmental health.

Overall, although multi‐omics and AI approaches hold promise for advancing MP health research, their application is currently limited by technical heterogeneity, data quality constraints, and insufficient standardization. These issues compromise data comparability, model robustness, and mechanistic interpretability, especially in high‐dimensional, small‐sample exposure contexts. Progress will require coordinated standardization, rigorous quality control, and phased integration of multi‐omics and AI into well‐designed prospective population studies.

## 7 Susceptible Populations and Differential Vulnerability

### 7.1 Infants and Older Adults: High‐Risk Groups Driven by Barrier Vulnerability

Infants experience relatively high exposure levels (e.g., extensive release of micro‐ and nanoplastics from polypropylene feeding bottles), and both the intestinal barrier and the BBB are still immature. These factors allow particles to more easily enter systemic circulation and brain tissue, while the underdeveloped immune system increases susceptibility to excessive ROS production and amplified inflammatory responses [199, 200, 201]. In contrast, older adults exhibit endothelial dysfunction, reduced antioxidant capacity, and elevated baseline inflammation, all of which compromise BBB stability. As a result, they are more vulnerable to MP‐induced downregulation of tight‐junction proteins, oxidative stress, and microvascular injury. Taken together, both age groups display heightened susceptibility due to physiological barrier fragility, immune vulnerability, and impaired inflammatory regulation.

### 7.2 Patients with Chronic Diseases: A Sensitivity Group Marked by Inflammatory Co‐Amplification

Individuals with chronic conditions—such as metabolic syndrome, diabetes, hypertension, and obesity—already exist in a state of low‐grade inflammation, high oxidative stress, and increased vascular permeability. These pathological features synergize strongly with MP‐induced ROS accumulation and NF‐κB/NLRP3 inflammasome activation. Hyperglycemia promotes AGEs accumulation and exacerbates BBB fragility [202]; hypertension‐induced shear stress abnormalities aggravate endothelial injury [203]; obesity‐related lipid dysregulation further amplifies MP‐induced inflammatory responses [204, 205]. Consequently, these populations are more likely to experience BBB disruption, impaired cerebral microcirculation, and intensified neuroinflammation under MP exposure, thereby elevating stroke risk.

### 7.3 Environmental Co‐Exposure and Genetic Background: Variable‐Risk Populations With Additive Susceptibility

In real‐world settings, MP exposure frequently co‐occurs with heavy metals, PAHs, PM2.5, and other pollutants. The formation of a “contaminant corona” on MP surfaces significantly enhances their barrier‐crossing ability and pro‐inflammatory potential, leading to exponentially amplified toxicity [197, 206]. Populations living in urban or industrial areas, or those chronically exposed to air pollution or psychological stress, may therefore exhibit increased neurovascular susceptibility. Furthermore, genetic polymorphisms in inflammatory regulatory genes (e.g., IL‐6, TNF‐α) or antioxidant pathways may result in pronounced inter‐individual differences in responses to MP exposure, giving rise to genetically susceptible subgroups. Thus, the health impact of MPs follows a multidimensional, heterogeneous risk pattern rather than being evenly distributed across populations.

Overall, the neurovascular risks associated with MP exposure are not uniform across populations but are shaped by multiple determinants, including barrier maturity, physiological and metabolic status, environmental co‐exposures, and genetic background. Infants and older adults are more vulnerable due to barrier fragility; patients with chronic diseases exhibit amplified responses due to underlying inflammation and metabolic dysfunction; and individuals exposed to high environmental pollution or carrying certain genotypes show increased sensitivity. These insights highlight the need for future health risk assessments and epidemiological studies to adopt a “differential susceptibility” perspective rather than assuming uniform risk. Building precision risk models centered on population vulnerability will be essential for developing more effective and targeted public health strategies.

## 8 Conclusion and Future Perspectives

Over the past decade, MPs have progressed from an “emerging contaminant” to a widely recognized environmental exposure of potential relevance to neurovascular health [207]. While early toxicological studies primarily focused on gastrointestinal, respiratory, and reproductive systems, accumulating experimental evidence suggests that MPs may influence neurovascular biology through alterations in gut and BBB integrity, inflammatory signaling, endothelial homeostasis, and cerebral microcirculation under specific experimental conditions. Animal and in vitro studies have reported cognitive impairment, endothelial dysfunction, neuroinflammation, and disruption of tight‐junction proteins following MP exposure [43, 77, 208]. However, these findings largely derive from controlled model systems and should be interpreted as indicating potential hazard rather than definitive evidence of human cerebrovascular risk.

Importantly, substantial uncertainties remain regarding MP biodistribution in humans, their capacity to cross the BBB, and the extent to which particle size, polymer composition, and surface properties govern biological effects. Epidemiological evidence linking MP exposure to cerebrovascular outcomes is limited and inconsistent, often indirect, and constrained by cross‐sectional designs, small sample sizes, and non‐standardized exposure assessment. Notably, a considerable number of studies conducted under environmentally relevant exposure conditions report minimal or no detectable neurovascular effects, underscoring the context‐dependent and heterogeneous nature of MP toxicity.

A major challenge in the field is the pronounced heterogeneity of MP materials and experimental models, including variability in polymer type, particle size, morphology, surface aging, and exposure paradigms. Together with differences in experimental platforms and dosing strategies, these factors limit cross‐study comparability and hinder direct extrapolation to real‐world human exposure scenarios. Consequently, the current evidence base should be viewed as hypothesis‐generating rather than conclusive.

In summary, while experimental studies support the biological plausibility that MPs may affect neurovascular function under certain conditions, definitive conclusions regarding human cerebrovascular risk cannot yet be drawn. Progress toward evidence‐based risk assessment will require standardized MP reference materials, harmonized exposure frameworks, improved human biomonitoring, and well‐designed prospective studies integrating mechanistic and epidemiological approaches.

Moving forward, several complementary research directions are essential.

First, standardized exposure models must be established. Chronic low‐dose ingestion paradigms incorporating quantitative MP burdens in blood, feces, and brain tissue will better reflect real‐world exposure conditions. Integrating these models with stroke paradigms and multimodal outcome measures (cerebral perfusion, endothelial function, neurobehavioral assessments) will clarify whether MPs exacerbate ischemic injury or hinder recovery.

Second, mechanistic dissection through multi‐omics is critical. High‐resolution platforms—including scRNA‐seq, proteomics, metabolomics, lipidomics, and spatial transcriptomics—can map cell‐type‐specific vulnerability in microglia, endothelial cells, and neural stem/progenitor populations following MP exposure [209, 210, 211]. Integrating multi‐omics across the gut–liver–brain axis may reveal molecular checkpoints and therapeutic targets that mediate MP‐induced neurovascular damage.

Third, AI‐driven predictive modeling offers powerful tools for risk assessment. Machine learning approaches integrating environmental exposure data, toxicological signatures, and clinical stroke registries could generate predictive models of exposure–biomarker–outcome relationships [212, 213]. Advanced frameworks such as graph neural networks may support nonlinear dose–response modeling, individualized risk stratification, and precision prevention strategies [214].

Finally, translation into public health practice is indispensable. Given the ubiquity of MPs, complete exposure elimination is unrealistic. Instead, regulatory thresholds in drinking water and food, improvements in purification technologies, and targeted screening of high‐risk populations should be prioritized. Emerging molecular biomarkers may be integrated into an “ environmental toxin–cerebrovascular health” risk scoring system to guide personalized intervention strategies.

Collectively, advancing standardized MP materials and exposure protocols, deep mechanistic profiling through multi‐omics, AI‐assisted risk prediction, and evidence‐based public health measures will be essential for elucidating the neurovascular consequences of MP exposure and establishing scientifically grounded safety thresholds for environmental plastic contaminants.

## Author Contributions

H.X.W, G.P., and X.Y.D wrote the main manuscript text and Y.J.H, P.Z, X.Y.W., and X.Y.D. prepared Figures 1, 2, 3, 4, 5, 6. All authors reviewed the manuscript.

## Funding

This study was funded by the National Natural Science Foundation of China Regional Science Fund Project (No. 82260455) and Joint Funding Project of the Hunan Provincial Natural Science Foundation and Hunan Xiangya Boai Rehabilitation Hospital Co Ltd. (No. 2025JJ90276).

## Conflicts of Interest

The authors declare no conflict of interest.
